# Supplementary material for: Acute coronary syndrome versus acute myocarditis in young adults–value of speckle tracking echocardiography
Source: PLoS One. 2022 Aug 8;17(8):e0271483. doi: 10.1371/journal.pone.0271483 (PMC9359587; doi:10.1371/journal.pone.0271483)
Supplement: S1 Table — (PDF) [file pone.0271483.s001.pdf]

Fig.	Mean (Myocarditis/MI)	Standard deviation (Myocarditis/MI)	Statistical method used	P value
Fig.1				
Endo/Epi ratio	2.5/2.3	0.6/0.7	Mann-Whitney U test	0.144
Fig.2b				
GLS Avg [%]	-18.7/-15.4	2.7/3.9	Mann-Whitney U test	0.000
Fig.5				
GCS Avg [%]	-17.5/-14.7	4.3/4.8	Mann-Whitney U test	0.036
Fig.6a				
GCS ENDO [%]	-25.3/-21.0	6.3/7.2	Mann-Whitney U test	0.009
Fig.6b				
GCS EPI [%]	-10.4/-8.8	3.1/3.3	Mann-Whitney U test	0.036

Fig.	Data used	Statistical method used	Results
Fig.2a	GLS Avg [%]	ROC analysis	AUC 0.737 (standard error 0.061, 95% confidence interval 0.795-0.954)
Fig.3a	GLS Avg [%]<br>LVEF [%]	Spearman's rank correlation coefficient	r=-0.7900<br>p=0.000
Fig.3b	GLS Avg [%]<br>LVEF [%] *	Spearman's rank correlation coefficient	r=-0.7695<br>p=0.000
Fig.3c	GLS Avg [%]<br>LVEF [%] **	Spearman's rank correlation coefficient	r=-0.7220<br>p=0.000

* Myocarditis

** Myocardial infarction

Data used	Numbers (Myocarditis/MI)	Percentages [%] (Myocarditis/MI)	Statistical method used	P value
Sex [male]	33/23	89.2/71.9	Chi-square test	0.012
Smoking	16/23	43.2/71.8	Chi-square test	0.017
Hypertension	5/15	13.5/46.9	Chi-square test	0.005
Diabetes	2/2	5.4/6.25	Chi-square test	0.713
Obesity	3/10	8.1/31.2	Chi-square test	0.032
Family history of CAD	11/11	29.7/34.4	Chi-square test	0.680
Chest pain on adm.	36/30	97.3/93.8	Chi-square test	0.898
Dyspnea	3/9	8.1/28.1	Chi-square test	0.062
**Data used**	**Mean (Myocarditis/MI)**	**Standard deviation (Myocarditis/MI)**	**Statistical method used**	**P value**
Age [years]	28/40	8/4	Mann-Whitney U test	0.00
HR [bpm]	82/83	13/14	Mann-Whitney U test	0.707
sBP [mmHg]	126/150	12/24	Mann-Whitney U test	0.000
dBP [mmHg]	76/91	10/22	Mann-Whitney U test	0.000

WBC	10.4/12.9	3.6/6.4	Mann-Whitney U test	0.108
RBC	4.9/5.0	0.4/0.5	Mann-Whitney U test	0.543
TnT on adm.	643/641	626/598	Mann-Whitney U test	0.021
TnT (after 24h)	932/2958	778/2742	Mann-Whitney U test	0.000
CKMB on adm.	31.6/29.8	32.8/48.0	Mann-Whitney U test	0.307
CKMB (after 24h)	43.2/140.3	50.2/167.0	Mann-Whitney U test	0.005
CRP	71.7/38.8	67.4/54.5	Mann-Whitney U test	0.006
PWd [mm]	10/11	1/2	Mann-Whitney U test	0.002
IVSd [mm]	10/12	1/2	Mann-Whitney U test	0.000
LVEDD [mm]	50/50	4/5	Mann-Whitney U test	0.538
LVESD [mm]	34/34	6/6	Mann-Whitney U test	0.976
LAVi [ml/m2]	30/30	5/12	Mann-Whitney U test	0.708
TAPSE [mm]	23/22	3/4	Mann-Whitney U test	0.182
LVEF [%]	56/50	7/8	Mann-Whitney U test	0.002
E/A ratio	1.5/1.2	0.3/0.3	Mann-Whitney U test	0.000
E/E’	7/8	2/2	Mann-Whitney U test	0.000
