# Supplementary material for: Acute coronary syndrome versus acute myocarditis in young adults–value of speckle tracking echocardiography
Source: PLoS One. 2022 Aug 8;17(8):e0271483. doi: 10.1371/journal.pone.0271483 (PMC9359587; doi:10.1371/journal.pone.0271483)
Supplement: Attachment — Submitted filename: Response to Reviewers.docx [file pone.0271483.s002.docx]

Dear Sir/Madam,

I would like to submit the revised version of the manuscript entitled “Acute coronary syndrome versus acute myocarditis in young adults – value of speckle tracking echocardiography” by Wieczorkiewicz P, Supel K, Przybylak K, Kacprzak M, Zielinska M to be considered for publication as an original article in the *PLOS ONE.*

On behalf of all co-authors, I would like to thank you very much for all the reviewers’ comments and suggestions.

Appreciating their input and effort, we have revised the manuscript in accordance with all the recommendations.

The first and foremost we have supplemented the statistics with the scatter plots of a strong correlation between GLS average and left ventricular ejection fraction in whole population and in both groups separately, what confirms the predictive role of GLS in whole analysis. The similar pattern of the correlations proves that this relation does not differentiate the groups.

In our study we have focused our attention on the differences between global and three – layers strains impairment in patients with acute coronary syndrome and myocarditis. Our results confirmed the characteristic ischaemic and non – ischaemic pattern of myocardial injury which is compatible with that observed in the CMR analysis. We do not consider the speckle tracking echocardiography as a differentiating tool between patients with ST segment elevation who should undergo coronary angiography or not, but it might constitute a valuable information to complement the diagnosis of myocarditis in patients with excluded CAD, especially when CMR is not easily available. We would like to also underlined that this study is the second part of our wider analysis of young patients with acute coronary syndrome versus myocarditis. We have evaluated the role of clinical, laboratory and ECG findings in this group and proposed the three – factor diagnostic model which was published 10 February 2022 in J. Clin. Med. [1]. Now, in order not to duplication the results we have concentrated mainly on the echocardiographic characteristic in this group of patients.

We have also rephrased the Conclusion paragraph and cited the recently published Position Statement by Seferovic et al.

Following further suggestions, we have added some information about STEMI localization and number of impaired LV segments on the basis of echocardiography examination in both groups. All patients diagnosed as ACS have undergone successful angioplasty of infarct-related artery.

As far as the differences in layer-specific strain impairment, indeed, we presume that the presence of mainly epicardial strain impairment in combination with preserved endocardial strain can be supportive for the myocarditis diagnosis. In our study, the mean value of endocardial GCS in MI patients (-21) is out of normal reference values for this age group which is (-28.4±3.3). Please, see Fig 6a. In GCS strains, the greater damage means values which are ‘less negative’. On the other hand, the lack of significant difference in endo/epi ratio between the groups might be the result of fast improvement of endocardial fibers in patients with STEMI undergoing primary PCI.

In our study, PWd and IVSd were significantly higher in myocardial infarction group probably due to the more cardiovascular risk factors including hypertension. In literature, there are several reports of LV wall thickening in acute myocarditis observed in echocardiography examination [2], but it occurs in only 18-50% of cases and is more marked in the fulminant course of the disease [3,4]. Interestingly, myocardial oedema, thanks to the CMR imaging, is observed also in patients with acute MI which corresponds to the ischemic area – at – risk [5-7].

Unfortunately, in our research we have not been able to complete the speckle tracking analysis with the right ventricle values.

We believe that these corrections will improve the manuscript in terms of its educational value.

We are looking forward to hearing from you at your earliest convenience.

Best regards,

Paulina Wieczorkiewicz

1. Wieczorkiewicz P, Przybylak K, Supel K, Kidawa M, Zielinska M. Symptomatic Young Adults with ST-Segment Elevation—Acute Coronary Syndrome or Myocarditis: The Three-Factor Diagnostic Model. Journal of Clinical Medicine 2022; 11(4), 916; <https://doi.org/10.3390/jcm11040916>.
2. Tiantian Xu, Hongjie H. A case report on transient global ventricular wall thickening secondary to acute myocarditis. Focus on the unique role of cardiac MRI. Medicine 2020;99:8(e19223).
3. Hiramitsu S, Morimoto S, Kato S et al. Significance of transient left ventricular wall thickening in acute lymphocytic myocarditis. Heart and Vessels 2007;22:25-29.
4. Nakagawa M, Hamaoka K. Myocardial thickening in children with acute myocarditis. Chest 1993 Dec;104(6):1676-8.
5. Gao H, Kadir K, Payne AR, Soraghan J, Berry C. Highly automatic quantification of myocardial oedema in patients with acute myocardial infarction using bright blood T2-weigted CMR. Journal of Cardiovascular Magnetic Resonance 2013;15:28.
6. Phrommintikul A, Abdel-Aty H, Schulz-Menger J, Friedrich MG, Taylor AJ. Acute oedema in the evaluation of microvascular reperfusion and myocardial salvage in reperfused myocardial infarction with cardiac magnetic resonance imaging. European Journal of Radiology 2010 Jun;74(3):e12-7.
7. Perazzolo Marra M, Lima JAC, Iliceto S. MRI in acute myocardial infarction. European Heart Journal 2011 Feb; 32(3):284-93.
